# Different Responses to Reward Comparisons by Three Primate Species

**DOI:** 10.1371/journal.pone.0076297

**Published:** 2013-10-09

**Authors:** Hani D. Freeman, Jennifer Sullivan, Lydia M. Hopper, Catherine F. Talbot, Andrea N. Holmes, Nancy Schultz-Darken, Lawrence E. Williams, Sarah F. Brosnan

**Affiliations:** 1 Language Research Center, Georgia State University, Atlanta, Georgia, United States of America; 2 Lester E. Fisher Center for the Study and Conservation of Apes, Lincoln Park Zoo, Chicago, Illinois, United States of America; 3 Wisconsin National Primate Research Center, University of Wisconsin, Madison, Wisconsin, United States of America; 4 Michale E. Keeling Center for Comparative Medicine and Research, UT MD Anderson Cancer Center, Bastrop, Texas, United States of America; 5 Department of Psychology, Philosophy & Neuroscience Institute, Georgia State University, Atlanta, Georgia, United States of America; Université de Strasbourg, France

## Abstract

**Background:**

Recently, much attention has been paid to the role of cooperative breeding in the evolution of behavior. In many measures, cooperative breeders are more prosocial than non-cooperatively breeding species, including being more likely to actively share food. This is hypothesized to be due to selective pressures specific to the interdependency characteristic of cooperatively breeding species. Given the high costs of finding a new mate, it has been proposed that cooperative breeders, unlike primates that cooperate in other contexts, should not respond negatively to unequal outcomes between themselves and their partner. However, in this context such pressures may extend beyond cooperative breeders to other species with pair-bonding and bi-parental care.

**Methods:**

Here we test the response of two New World primate species with different parental strategies to unequal outcomes in both individual and social contrast conditions. One species tested was a cooperative breeder (*Callithrix* spp.) and the second practiced bi-parental care (*Aotus* spp.). Additionally, to verify our procedure, we tested a third confamilial species that shows no such interdependence but does respond to individual (but not social) contrast (*Saimiri* spp.). We tested all three genera using an established inequity paradigm in which individuals in a pair took turns to gain rewards that sometimes differed from those of their partners.

**Conclusions:**

None of the three species tested responded negatively to inequitable outcomes in this experimental context. Importantly, the *Saimiri* spp responded to individual contrast, as in earlier studies, validating our procedure. When these data are considered in relation to previous studies investigating responses to inequity in primates, they indicate that one aspect of cooperative breeding, pair-bonding or bi-parental care, may influence the evolution of these behaviors. These results emphasize the need to study a variety of species to gain insight in to how decision-making may vary across social structures.

## Introduction

Many aspects of social cognition and behavior are sensitive to both ecological and social factors. A good example is cooperation, which is affected by a number of factors, including the identity of the partner, the reward distribution and information about the partner’s previous decisions [1], [2]. In particular, a species’ social structure should have profound implications for the evolution of social behavior and a recent hypothesis - the cooperative breeding hypothesis - formalizes this in the case of those species that work together with relatives to raise offspring [3], [4]. So-called ‘cooperative breeders’ have an unusually high level of interdependence because offspring survival depends on both parents and a number of additional helpers to provide alloparental care [5], [6]. It is proposed that these species should show more prosocial behavior to non-kin than do non-cooperative breeders, even those that cooperate in other contexts, due to selective pressures related to cooperative breeding [3], [4], and evidence indicates that this is the case. Tamarins and marmosets, two cooperatively breeding primates in the family Callithrichidae, are more likely than many other non-human primate species to provide food to their breeding partners in tests of prosocial behavior ([7], [8]; but see [9], [10] for contrary evidence in Callithrichids and [11], [12] for reviews of evidence that other species, too, show this behavior). Moreover, it has been argued that sharing amongst cooperative breeders more often involves the active sharing of food, in which the possessor gives food to the recipient, as opposed to the passive sharing of food (e.g., sharing initiated by the recipient) more often seen in non-cooperative breeders [11].

Brosnan [13] recently proposed that the same factors that increase prosocial behavior in cooperative breeders should also influence these species’ responses to inequitable outcomes (i.e. social contrast). It may be that cooperative breeders are highly sensitive to inequity because of their high rates of cooperation. Alternatively, their high levels of interdependence, which increase prosocial behavior, may also make them less likely to respond to minor inequities. However, cooperative breeding is not the only situation in which interdependence occurs; some primate species are pair-bonded and show bi-parental care, but are nevertheless not classified as cooperative breeders. If the proposed suite of cognitive and behavioral adaptations for cooperative breeding is essential, one would anticipate that these species would behave differently than other species who are also interdependent, but lack these specific adaptations for cooperative breeding. On the other hand, it may be that pair-bonding and bi-parental care are both sufficient to elicit these social behaviors, in which case both cooperative breeders and those showing bi-parental care would show similar responses to inequity. We contrasted these hypotheses by examining responses to inequity in two New World primate species with bi-parental care, one of which is classified as a cooperative breeder. These data can be compared to those from other primate species that are not cooperative breeders [13] as well as one that is [14] to see whether these monkeys’ outcomes support this hypothesis. Additionally, to verify that the slightly modified procedure that we employed was comparable to that used in previous primate studies of inequity, we tested a third confamilial New World primate (*Saimiri* spp.) to compare its responses between the original and modified procedures.

For the current study, we used an adaptation of the established exchange paradigm for measuring responses to inequity. This paradigm has been used by a variety of researchers to test both primate and non-primate species’ responses to unequal outcomes (for a review see [13], [15]). Our results, therefore, provide continuity with the existing literature and a strong comparison across the primates. Importantly for our purposes, previous data demonstrate that this response varies amongst the primates, indicating that selective pressures have altered it since the common ancestor to the primates. Among the great apes, chimpanzees often respond negatively to inequity (*Pan troglodytes*; [16], [17]; but see [18]), while another ape, the orangutan (*Pongo pygmaeus*), does not [18], [19]. In Old World monkeys, both long-tailed macaques (*Macaca fasciularis)* and two year old rhesus macaques (*Macaca mulatta*) respond negatively to inequity [20], [21]. Among the New World monkeys, capuchins routinely respond negatively in this task (*Cebus apella*; [22], [23], [24]; but see [25]) while, the con-familial squirrel monkey, does not (*Saimiri spp*.; [26]). However, squirrel monkeys were not insensitive to the procedure; they responded to a control designed to test for contrast effects ([27], i.e. a violation of individual expectations, rather than social comparison, [26]). Unlike capuchin monkeys, squirrel monkey males (but not females) responded to this contrast condition. Thus, there is an interesting divergence within the New World monkeys, with capuchins responding to social comparison (i.e. inequity), but not individual contrast effects, while squirrel monkeys do the opposite. There has thus far been only one test of inequity on any cooperative breeder, cotton top tamarins (*Saguinus oedipus*), with equivocal results; subjects did not appear to respond to unequal outcomes, but did respond differently between the conditions requiring subjects to work for their rewards and those simply handing out those rewards for free [14].

Both common marmosets and owl monkeys are New World monkey species, and are thus phylogenetically close to capuchin and squirrel monkeys, as well as tamarins. Marmosets live in groups ranging from three to 15 individuals, consisting of dominant breeders and adult family members that help care for infants in the group [5]. The non-breeding adult females are often anovulatory and instead of leaving to form their own breeding groups they stay with the group and help to raise infants [5]. Owl monkeys live in small groups of two to five individuals, typically consisting of a male and female and their offspring [28]. Critically for our test, owl monkeys engage in bi-parental care such that both parents are essential for the survival of the offspring, but are not considered cooperative breeders [28].

In previous inequity studies involving monkeys [22], [24], the pair was separated by a barrier that allowed individuals to see each other and interact, but this meant that they did not share the same space. Due to the pair-housing of the marmosets and owl monkeys, they were tested without separation (as has been done in some ape studies; [16], [17], [19]). In order to avoid the possibility that this change in procedure influenced the monkeys’ responses, we re-tested squirrel monkeys without separating them to serve as a control and to verify that their behavior did not change in this situation in comparison to when tested with a barrier separating them [26]. Additionally, we chose to use a targeting task with the marmosets and owl monkeys (rather than the typically-used exchange task [e.g., 26]), so the same targeting task was also used with the squirrel monkeys to verify that the important element was the addition of a task, not the exchange task *per se* [31]. Although squirrel monkeys do not themselves respond to inequity, we chose this species as they were housed at the same facility as the owl monkeys, which minimized the possibility that housing or husbandry differences would underlie any observed differences in behavior shown by the monkeys.

While squirrel monkeys do not respond to inequity they do, as mentioned above, respond negatively to contrast effects, thus there was variation that would presumably allow us to detect if there were differences between conditions. The proposed reason behind squirrel monkey’s lack of response to inequity is different than that that of the marmosets or owl monkeys. Currently, the pattern of the data best fit the hypothesis that species that cooperate with non-kin also respond to inequity in these experiments [13], [34]). It has been proposed that responding to inequity allows individuals to identify cooperative partners who are taking more than their share and try to find a new one, a behavior which is likely to, on average, increase the benefit to cooperation [32], [15]. Squirrel monkeys (along with orangutans, which also do not respond to inequity) only cooperate in limited contexts and therefore may have less need to pay attention to a partner’s outcomes [13]. Thus the proposed link between cooperation and inequity is two-fold First, species who cooperate often in non-breeding contexts, such as chimpanzees, capuchins, bonobos, and macaques all respond to inequity, presumably because of the benefits of identifying good partners for cooperation. These species may use this information to determine when to continue working with a partner and when to cease cooperating and search for a new potential partner. Second, cooperative breeders (and possibly bi-parental care species) cooperate extensively, but to the extent that their fitness is dependent upon their partner. This degree of interdependence changes the calculus such that it may be *against* their best interests to respond negatively to a situation of inequity as the costs of finding a new partner are extreme [13]. This study focused on the second part of this hypothesis, and explored the behavior of species which cooperate in breeding contexts, either as cooperative breeders (i.e. marmosets) or in a bi-parental care context (i.e. owl monkeys). Understanding their responses to inequity will help to build a broader picture for understanding responses to inequity in the context of cooperation.

Our study included four conditions. To measure responses to inequity, we compared how subjects responded when their partner got a more preferred reward than they did (Inequity Baseline, IB) to when they and their partner got the same less-preferred reward (Equity Control, EC). To test whether these species respond to individual contrast (that is, to a comparison with a previously offered reward [33]), we included a condition in which their attention was drawn to their preferred reward prior to completing the task but were then offered a less preferred option (High-value Reward Control, HRC). Both rewards were visible at all times in all conditions. Finally, in other primates, subjects respond differently to unequal rewards that were ‘earned’ for completing a task versus those that were obtained for free [14], [16], [26], [31], [34]. To test whether or not these species, too, respond differently in the presence or absence of a task, we included a ‘Gift Reward’ condition (GR) in which subjects were given different rewards for free, that is, without first requiring the targeting task.

We hypothesized that, if cooperative breeding provided key adaptations that influenced these social behaviors, marmosets would not respond to inequity while owl monkeys would do so. If, on the other hand, neither owl monkeys nor marmosets responded to inequity as compared to the other conditions, as is true in tamarins [14], this would contrast with the responses of primates that cooperate with non-kin in other contexts (e.g., capuchins and chimpanzees). One possible explanation for this would be the importance of pair-bonding and bi-parental care, even in the absence of cooperative breeding. We did not have a specific prediction about these species’ responses to individual contrast, but included the condition to verify that any responses to “inequity” required a social partner to receive a different outcome. Finally, we predicted that squirrel monkeys would behave as in previous studies, despite the new procedure, with males responding to individual contrast, but not to inequity [26].

## Methods

### Ethics Statement

This research was conducted at the Michale E. Keeling Center for Comparative Medicine and Research, UT MD Anderson Cancer Center, Bastrop, TX, USA (KCCMR) and the Wisconsin National Primate Research Center, Madison, WI, USA (WNPRC) both of which are fully accredited by AAALAC-I (Common marmosets G00579-05-02-2009; Owl Monkeys 03-09-02681; Squirrel monkeys 03-09-02781). All animals were tested in their home cages and participated voluntarily in all tests. All subjects had ad libitum access to primate chow and water. At no time were the subjects ever food or water deprived. Subjects were supplemented daily with fruit and/or vegetable food enrichment. We thank the animal care and enrichment staff for maintaining the health and wellbeing of the monkeys. All procedures used in the research are in accordance with the Guidelines for the Use of Animals in Research and have been approved by the Institutional Animal Care and Use Committee of the Keeling Center for Comparative Medicine and Research of The University Texas M. D. Anderson Cancer Center (IACUC protocol 04-07-03682) and of the Wisconsin National Primate Research Center (IACUC protocol G00509).

### Subjects

Ten adult marmosets (*Callithrix jacchus*; 5 mated pairs; mean age = 4.7 years), were tested in their home cages at WNPRC. Eight adult owl monkeys (*Aotus spp.;* 2 mated pairs, 1 male pair and one mother/son pair; mean age = 5.3) and 14 squirrel monkeys (*Saimiri boliviensis*; 4 male-male pairs; mean age = 1.8 years; 3 male-female pairs; mean age = 1.0 years) were tested in their home cages at KCCMR. None of the subjects of any species had been previously exposed to studies involving inequity or cooperation. Subjects were all tested with individuals from their established social group. As owl monkeys are a nocturnal species [28], we tested them during their ‘dark cycle’ when they were at their most active (this was in red-light conditions so that the experimenters could see), while the squirrel monkeys and marmosets were tested during daylight hours.

### Procedure: Food Preference Tests

Prior to testing, food preferences were determined using a dichotomous-choice test to establish a high-value reward (HVR) and a medium-value reward (MVR; [22]), the latter of which are preferred foods for which the monkeys were willing to work, but were not as preferred as the HVRs (see criteria below). Each session consisted of 10 consecutive trials in which the experimenter held up an HVR in the palm of one hand and a MVR in the other, approximately 15 cm apart, centered on the monkey. Subjects indicated their choice by reaching their hand through the mesh and taking the preferred food. Subjects could also indicate preference by placing their mouth next to the reward they wish to receive (e.g., for liquid rewards; see below). To control for any side biases, presentation of the rewards alternated each trial between left and right. Subjects had to prefer the HVR to the MVR at least 80 percent of the time in two sessions run on different days and, in a separate session, eat 10 consecutive pieces of the MVR. Food rewards were only used if both subjects in a pair passed both preference tests, and food rewards did not change over the course of testing. Both individuals in a pair always used the same HVR and MVR, however, food rewards sometimes differed between different pairs to reflect different individuals’ preferences (see SOM for details).

### Procedure: Training

Prior to testing, all subjects were trained to do a ‘targeting’ task, which was the same for all species tested. All monkeys were required to reach for, and pull into their cage, an inedible plastic token. All training followed a positive reinforcement shaping procedure [29], [30] tailored for each species. Success resulted in a food reward (but one different from the rewards given in test sessions).

For the marmosets, tokens consisted of hollow, hard plastic tubing (polyethylene), approximately 5.08 cm in length and.64 cm in diameter. Subjects were considered proficient when they pulled the token in and held it for 1 second on at least 75% of trials on two consecutive daily training sessions. For the owl monkeys and squirrel monkeys, tokens consisted of hard plastic tubing, 5.08 cm in length and 1.27 cm in diameter. Subjects were considered proficient at the task when they pulled it in and held for at least 1 second on at least 75% of trials on two consecutive daily training sessions.

### Procedure: Testing

Pairs of all three species were tested together in their home cage (e.g., they were not separated from one another). Accordingly, subjects could easily observe what their cage-mate was exchanging and which reward they received during these interactions. Two reward containers (one for the MVR and one for the HVR) were always present, full, and in the same position, regardless of whether they were used in the session, so that the presence of either of these rewards did not cue the subject or create differences in reactions. Responses were immediately recorded on data sheets by the experimenter and all test sessions were videotaped for later analysis and coding.

All species were tested following the same protocol save for minor alterations as dictated by either the species (i.e., owl monkeys were tested during their night-time period, rewards were chosen based on species preferences; see Procedure: Food Preference Tests, above) or the facility where they were tested (i.e. KCCMR *versus* WNPRC; see SOM for details). A previous study using New World monkeys at different facilities found no significant differences in subjects’ behavior on the same protocol simply due to subjects being housed at different facilities [31]. As different experimenters tested the three species (H.F. tested owl monkeys, J.S. tested marmosets and L.H. and C.T. tested squirrel monkeys), to ensure inter-tester reliability, the senior author visited each site to ensure consistency among all procedures, and experimenters shared videos of their procedures before each new species was tested. Given this rigorous protocol, our careful training, and our extensive control tests involving the squirrel monkeys, we believe that differences in the species’ responses represent species-specific differences, rather than methodological differences arising from testing occurring at different facilities.

For each species, we tested the maximum number of trials that they would reliably complete. All species were supposed to receive 20 trials per monkey, and marmosets, who were tested first, received 40 trials alternating between the partner and the subject, such that each individual completed 20 trials per session. However in pilot testing, we found that the owl monkeys would not eat 20 pieces of food in a row, so we reduced the number of trials to 15 per monkey. To assure that responses were equivalent, we compared the responses of the marmosets in the first 15 trials for each marmoset to all 20 trials and found no significant differences (for each condition, the average percent-refusals for their first 15 trials and all 20 trials respectively were: HRC 8.7 and 9.3; EC 11.7 and 14.5; IB 8.7 and 9.3; GR 1.0 and 0.8; all *p* values>0.05). As there was no difference between their first 15 responses compared to all 20, we report all 20 trials in subsequent analyses below for completeness. Squirrel monkeys also received only 30 trials per session (15 apiece). Subjects participated in two sessions of each of the four conditions in the subject role (see below for details).

The order of sessions was randomized for each pair. The partner always completed the task prior to the subject. Time between trials was approximately 5 seconds, which was the amount of time it took the experimenter to record the results and prepare for the next trial. In each trial, the monkey had up to 10 seconds to respond to the token. After a successful trial, the experimenter lifted the predetermined reward corresponding to the trial from the container, placed it in the palm of her hand, raised it up in the front of the monkey (but out of reach) so that it was visible to both monkeys, and then gave the reward to the monkey who had just completed the task. Subjects could refuse to complete the task or refuse to accept the food reward. Ignoring the token, pushing the token away (rejecting), or pulling the token in only part way and then stopping without resuming within ten seconds were considered refusals to complete the task. Refusals to accept the reward consisted of ignoring it, throwing it away, or refusing to accept it.

### Coding and Analysis

To test whether the monkeys responded when their cage-mate received a lower-value reward (MVR), we compared subjects’ reactions in the Inequity Baseline (IB) to the Equity Control (EC). In the IB, both monkeys had to exchange; however, the subject received a MVR and the partner received an HVR. In the EC, both monkeys exchanged for an MVR. To determine whether the subject’s response was due to the partner getting a better reward (social contrast) or frustration over not receiving a better reward that appeared to be available (individual contrast), we compared the IB to the High-value Reward Control (HRC), in which both monkeys were shown a HVR prior to exchange, but after completing the exchange received a MVR. To test the hypothesis that the inclusion of a task elicits a different response, we compared the IB to the Gift Reward (GR), in which both individuals received their respective reward (subject MVR, partner HVR) for ‘free’, without having to complete the task beforehand. Only three of the seven squirrel monkey pairs received the GR condition as their response to the task was our focus in including them as a control species. Note that we had previously demonstrated that squirrel monkeys do not respond in a GR condition (they refused on less than 10% of trials, considerably less than the 22–50% refusals in experimental conditions, [26]).

All comparisons used the overall refusal rate (combining refusal to participate in the targeting behavior with refusals of the reward). Due to the small sample size, all statistics are non parametric. Overall comparisons were done with Friedman’s Tests, and paired comparisons with Wilcoxon Sign-Rank exact tests. Cross-species comparisons were made with Mann Whitney U tests for unrelated samples. All p values are two-tailed, and significance was considered to be p<0.05. To correct for potential family-wise errors, for all post-hoc pairwise analysis, we applied a Holm’s sequential Bonferroni correction [35]. We had a second coder, blind to our hypotheses and the conditions, code 20% of trials for all three species and found very high reliability on whether a trial was completed or not by the subject (owl monkeys: κ = 0.913; squirrel monkeys, κ = 0.938; marmosets, κ = 0.956).

## Results

### Individual Species’ Responses

**Figure 1:**
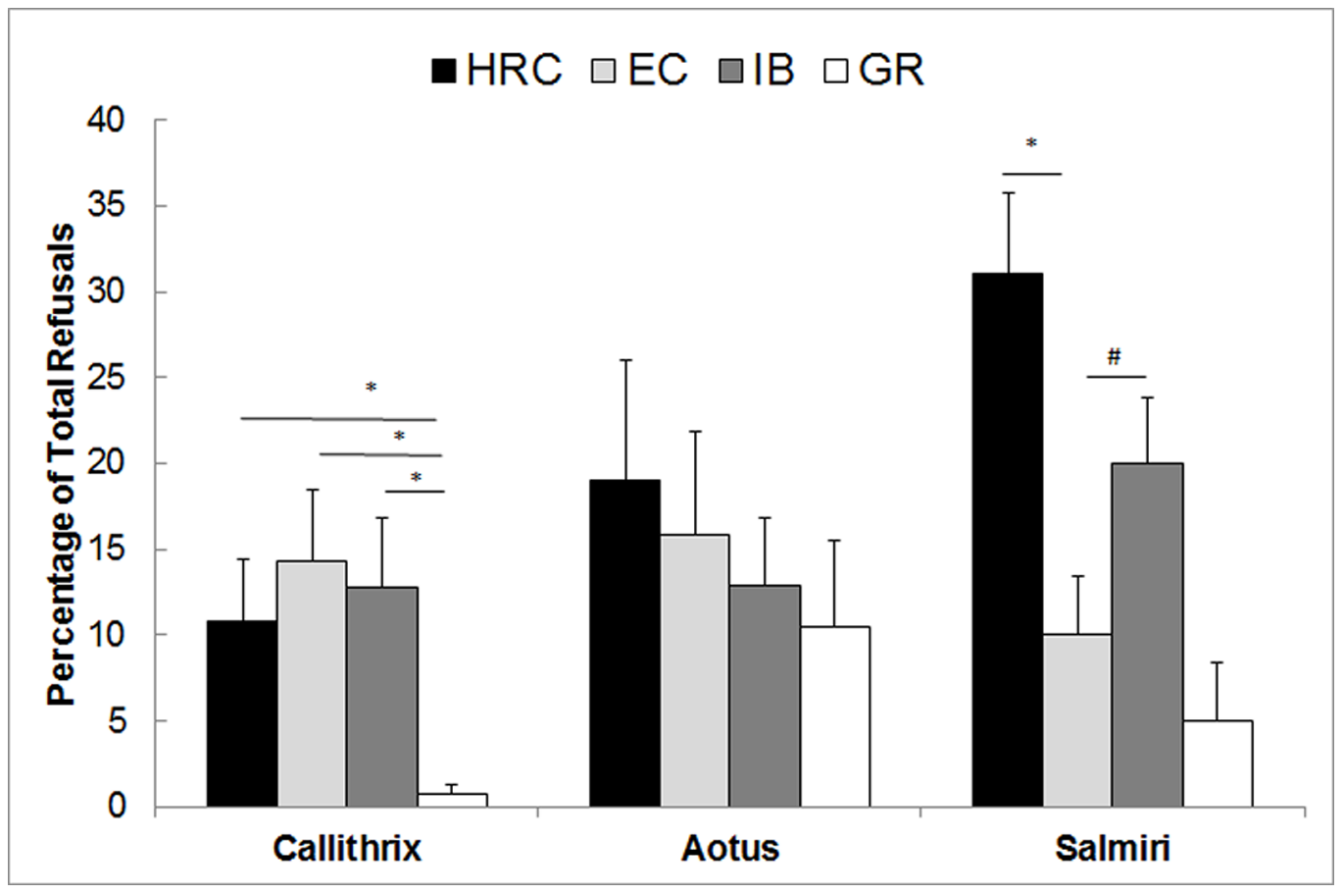
Percentage of refusals (showing standard error of the mean for each) in each condition for the three.

Marmosets varied in their rate of refusal among the four conditions of IB, HRC, EC and GR (Friedman’s Test, n = 10, χ^2^ = 19.18, df = 3, *p*<0.001, Figure 1). However, when the non-task based GR condition was excluded from analyses, marmosets did not differ in their rate of refusal among the other three conditions (IB, HRC, and EC; Friedman’s Test, n = 10, χ^2^ = .58, df = 2, p = 0.75). This did not change when food and token refusals were considered separately (comparing IB, HRC, and EC token refusals; Friedman’s Test, n = 10, χ^2^ = .16, df = 2, p = 0.9; food refusals; Friedman’s Test, n = 10, χ^2^ = .20, df = 2, p = 0.4) Thus, their rate of refusal did not differ depending on the value of their reward with respect to either their partner’s reward (IB) or the presence of a higher-value reward in the environment (HRC). As with previous work in other primates, marmosets refused far less often in the GR, when rewards were handed out for free, than in any other conditions (Wilcoxon Signed-ranks tests; all *p*s<0.001).

Owl monkeys did not differ in their rate of refusals among the four conditions (that is, IB, HRC, EC and GR; Figure 1; Friedman’s test, n = 8, χ^2^ = 3.61, df = 3, p = 0.31). This did not change when food and token refusals were considered separately (token refusals; Friedman’s Test, n = 8, χ^2^ = 4.16, df = 2, p = 0.1; food refusals; Friedman’s Test, n = 8, χ^2^ = 6.3, df = 3, p = 0.1).

Finally, verifying that our adapted procedure replicated previous such research with squirrel monkeys [26], the squirrel monkeys in this study also varied in their rate of refusal among the three conditions involving a task (IB, HRC, and EC, Figure 1; Friedman’s Test, n = 14, χ^2^ = 13.74, df = 2, *p = *0.001). As in Talbot et al. [26], we found that the squirrel monkeys showed increased refusals in the HRC condition as compared to the EC (Wilcoxon Signed Ranks test: *T = *0.0, n = 13, *p* = 0.001). The monkeys showed no differences in their refusals in the IB compared to the EC (*T = *18.0, n = 13, *p* = 0.054) nor between the HRC and IB (*T = *24.5, n = 13, *p* = 0.142). Again as in [14], the response to the HRC was driven by the males (comparing HRC to EC: *T = *0.0, n = 10, *p* = 0.003; females showed no variability across the conditions: n = 3, χ^2^ = 1.64, df = 3, *p = *0.441). This was true for both male-male pairs *T = *0.0, n = 8, *p* = 0.012) and males in male-female pairs (*T = *0.0, n = 6, *p* = 0.041).

### Cross-species Comparisons

To verify that the marmosets’ and, in particular, the owl monkeys’ responses were not dissimilar from those seen in species that do show variability across these different conditions, which might indicate a difference in their understanding of the task, we compared their refusal rates to both each other and to squirrel monkeys, our control species. The marmosets refused less than the owl monkeys in the GR (Mann Whitney U: *U* = 113.0, N_Callithrix_ = 10, N_Aotus_ = 8, *p = *0.040). There was no difference, however, in their refusals across the other three conditions: EC (*U* = 165.0, N_Callithrix_ = 10, N_Aotus_ = 8, *p = *0.87), IB (*U = *116.0, N_Callithrix_ = 10, N_Aotus_ = 8, *p* = 0.39), and HRC (*U = *132.0, N_Callithrix_ = 10, N_Aotus_ = 8, *p = *0.36).

The marmosets and squirrel monkeys showed comparably low levels of refusals in the IB (*U = *211.0, N_Callithrix_ = 10, N_Saimiri_ = 14, *p = *0.143), EC (*U = *349.0, N_Callithrix_ = 10, N_Saimiri_ = 14, *p = *0.129), and GR (*U = *100.0, N_Callithrix_ = 10, N_Saimiri_ = 14, *p = *0.454). In contrast, the squirrel monkeys refused more than the marmosets in the HRC (*U = *120.0, N_Callithrix_ = 10, N_Saimiri_ = 14, *p*<0.001). Similarly, the owl monkeys and squirrel monkeys showed comparably low levels of refusals in the IB (*U = *169.5, N_Aotus_ = 8, N_Saimiri_ = 14, *p = *0.175), EC (*U = *263.5, N_Aotus_ = 8, N_Saimiri_ = 14, *p = *0.297), and GR (*U = *108.5, N_Aotus_ = 8, N_Saimiri_ = 14, *p = *0.568). As compared to the marmosets, the squirrel monkeys refused more than the owl monkeys in the HRC (*U = *139.5, N_Aotus_ = 8, N_Saimiri_ = 14, *p = *0.037). We also compared the refusals made by the squirrel monkeys tested with this present task (‘target’) to those male squirrel monkeys we had tested previously with the exchange protocol (‘exchange’, see [26] for details of the methods used). As we tested both male and female squirrel monkeys, we compared our data to the responses of both the male and female monkeys tested by Talbot et al. [26]. There was no difference in the number of refusals made by monkeys when tested with the target *versus* the exchange procedure in either IB (*U = *511.5, N_target_ = 14, N_exchange_ = 24, *p* = 0.083) or HVR (*U = *563.0, N_target_ = 14, N_exchange_ = 24, *p* = 0.240). Those squirrel monkeys tested previously by Talbot and colleagues [26], however, made nearly three times more refusals in the EC (average = 29.0%) when tested with the exchange procedure than did the monkeys tested in the same condition in the present study with the target procedure (average = 9.8%; *U = *320.5, N_target_ = 14, N_exchange_ = 24, *p*<0.001).

## Discussion

In the current study, we examined responses to inequity, a behavior that may be related to the degree of interdependency and the degree of prosocial behavior, by comparing two species previously untested with this paradigm. Specifically, we compared the responses of a cooperative breeder (*Callithrix,* marmosets) with a non-cooperative breeder that nonetheless shows pair-bonding and bi-parental care (*Aotus,* owl monkeys). This provided insight into how the behavioral and cognitive adaptations cited by the cooperative breeding hypothesis [4], [34] influence marmosets’ reactions to unequal and unexpected outcomes and whether pair-bonding and bi-parental care might also select for a similar suite of adaptations. In support of the latter, neither marmosets nor owl monkeys responded negatively to a situation in which their partner received a higher value reward for completing an experimental task, as compared to control conditions. As predicted, the squirrel monkeys responded as in previous studies, verifying that our procedural changes did not influence outcomes in their responses to these experimental conditions.

A previous hypothesis proposed that amongst species that share parental care duties, the costs of responding negatively to inequity may outweigh the benefits [13]. That is, given the interdependent nature of their relationships, the cost of having conflict with their reproductive partner or, for cooperative breeders, with social group mates, may be too high to merit reaction over a small amount of inequity, such as created in this experimental paradigm. Such cost-benefit evaluations are seen in other social situations as well; tamarins are among the very few primate species that do not show reconciliation, which is argued to be because a small amount of aggression does not damage their relationships in the same ways as is the case in other species [36]. Aside from providing evidence that the demands of cooperative breeding may have selected for changes beyond increased prosocial behavior, these results also demonstrate the impact of interdependence on non-cooperative breeders who nonetheless show bi-parental care.

Of course, marmosets, tamarins, and owl monkeys are not the only primates to fail to respond to inequity; both orangutans and squirrel monkeys do as well, as evidenced by the squirrel monkeys used as the control species in this study. Currently, the most parsimonious explanation for these species’ responses (or lack thereof) seems to be that the failures to respond negatively to inequity have two different causes. If, as is hypothesized, responding negatively to inequity allows individuals to identify good cooperative partners or to find a new partner if they are not benefitting from the cooperative interaction, then individuals which are interdependent, such as in a mated pair, should be less likely to respond to inequity because the costs of finding a new partner are greater than the momentary inequity. This is consistent with our results for marmosets and owl monkeys, as well as previous results for tamarins [14]. This also means that species that cooperate often in contexts such as obtaining food, but not in the context of shared parenting, should respond to inequity, and this is what is seen in other primates [chimpanzees, bonobos, capuchins and macaques: 18, 20, 21, 22, 24]. On the other hand, for those species that do not regularly cooperate, there may not have been evolutionary pressure to recognize, or respond to, unequal outcomes, because there was far less need to identify good (or bad) cooperative partners.

This may explain the finding that squirrel monkeys and orangutans, who do not regularly cooperate, do not respond to inequity [13], [26]. Additional data are needed to explore this further. In particular, much would be learned from testing this hypothesis on other taxa that show variation in breeding systems, including both cooperative breeders and non-cooperative breeders. This could be done in non-primate mammals, such as meerkats, as well as with fish and bird species. Although some work is underway to explore inequity responses in both fish and birds [37], [38], additional work is needed to test this hypothesis.

It is interesting to consider how these results compare to earlier ones finding that relationship quality affects inequity outcomes within specific relationships in humans [39] and, possibly, chimpanzees [16]. Although cooperative breeding, overall, selected for a suite of behaviors in cooperative breeders, the manifestation of these responses may vary depending upon the quality of the relationship. To date, data indicate that these responses may be consistent across several types of relationships. For instance, in our data, the one owl monkey pair that was neither a breeding pair nor a mother/offspring pair responded in the same way as the other pairs. This is similar to a previous result that found that common marmosets behaved equally prosocially with their mated pair and with other members of their family group [7]. However, in all of these cases, the partner was still another adult with whom the monkey was co-housed, and thus the partners had developed a relationship. It would be useful to test relationships at different time points to see how this impacts their responses. For example, humans are often considered to be cooperative breeders [39], but unusually amongst primate cooperative breeders, we also interact extensively outside of our mated pair. The majority of experimental decision-making studies (such as in psychology or experimental economics) test strangers or anonymous pairs, rather than individuals with existing relationships. It would be useful to investigate how human decision-making varies across these relationship contexts. We look forward to future work that untangles the effect of selection to support members of the family group and the (possibly collateral) effect of relationship quality on behavior.

We also note that, despite a similar tendency to tolerate inequitable outcomes, there were differences in the response between marmosets and owl monkeys. In particular, the marmosets were sensitive to the presence of a task, responding more often when they had to complete a task to receive the reward than when they were handed it for free, while the owl monkeys were not. That is, the marmosets were more likely to refuse rewards when they had to work to receive the rewards, as has been shown previously for squirrel monkeys [26]. However, unlike squirrel monkeys, marmosets’ responses did not vary across the other conditions that involved the task, indicating that while this response may be about expectations for payoffs, it is unrelated to the equity, or lack thereof, of the interaction. Considered with results from tamarins [14], it appears that Callitrichids join other primates in showing sensitivity to the presence of a task [13], [16], [17], [26], [31], if not the equity of their outcomes. Owl monkeys, however, responded to the 'free' gift reward condition similarly to the other conditions requiring effort on their part (see also [40]). Although we cannot rule out that the owl monkeys and marmosets simply did not notice their partner’s reward, owl monkeys and marmosets do beg for food from conspecifics [40], indicating that they are attentive to others and to food items potentially available to them in their environment.

One of our critical comparisons involved the squirrel monkeys, a species for whom responses to individual contrast, in which individuals refuse more often after having been offered a higher-value reward, has previously been documented [26]. We were able to verify that in this protocol, which differed from Talbot and colleagues’ [26] both by the use of a targeting, rather than exchange, task and the fact that the monkeys were not separated by a mesh partition, the results were the same; in both cases, squirrel monkey males responded to individual contrast. These results show that while a task is apparently critical in eliciting individual or social contrast [17], the form that this task takes is flexible [see also 21]. This may make it easier to test species for which exchange is not possible, using an analogous paradigm [e.g. 38]. We also note that of the previous species studied in inequity research, squirrel monkeys are the only species who only responds to individual contrasts, but not to inequity. Further research is needed to understand why that might be the case.

We found evidence supporting the hypothesis that a social structure with pair-bonding and bi-parental care, not just cooperative breeding, impacts responses to inequity in primates [16]. We look forward to additional research, both in primates and other taxa, that explore this question in more depth. Although we know that cognition, including social cognition, varies depending upon a species’ ecology [2], [41], [42] and relationships [4], [43], these results extend this by providing support for being mindful of the selective pressures due to a species’ social environment when considering questions of social cognition. Thus these results join the growing body of literature indicating the importance of phylogenetic comparisons to test evolutionary hypotheses regarding the origins of social cognition and behavior [44].
